# Mass gatherings: a review of the scope for meningococcal vaccination in the Indian context

**DOI:** 10.1080/21645515.2020.1871572

**Published:** 2021-02-19

**Authors:** Anand P Dubey, Rashna Dass Hazarika, Veronique Abitbol, Shafi Kolhapure, Someya Agrawal

**Affiliations:** a Pediatrics, ESI-PGIMSR & Model Hospital, New Delhi, India; b Pediatrics, Nemcare Superspeciality Hospital, Bhangagarh, Guwahati, and RIGPA Children’s Clinic, Guwahati, India; c GSK, Rueil-Malmaison, France; d GSK, Mumbai, India; e GSK, New Delhi, India

**Keywords:** Crowd, IMD, India, mass gathering, meningococcal disease, *Neisseria meningitidis*, outbreak, prevention, travel, vaccination

## Abstract

The risk of meningococcal transmission is increased with crowding and prolonged close proximity between people. There have been numerous invasive meningococcal disease (IMD) outbreaks associated with mass gatherings and other overcrowded situations, including cramped accommodation, such as student and military housing, and refugee camps. In these conditions, IMD outbreaks predominantly affect adolescents and young adults. In this narrative review, we examine the situation in India, where the burden of IMD-related complications is significant but the reported background incidence of IMD is low. However, active surveillance for meningococcal disease is suboptimal and laboratory confirmation of meningococcal strain is near absent, especially in non-outbreak periods. IMD risk factors are prevalent, including frequent mass gatherings and overcrowding combined with a demographically young population. Since overcrowded situations are generally unavoidable, the way forward relies on preventive measures. More widespread meningococcal vaccination and strengthened disease surveillance are likely to be key to this approach.

## Introduction

The World Health Organization (WHO) has described mass gatherings as events “characterized by the concentration of people at a specific location for a specific purpose over a set period of time and which has the potential to strain the planning and response resources of the country or community”.^1^ While much of the literature describes gatherings exceeding 25,000 persons, a mass gathering can be as few as 1,000 people.^1^ This could be an organized occasion, such as a social function, sports competition, or political, religious, or cultural gathering, or it may occur spontaneously, for example, in association with a funeral, social unrest, or social upheaval. The duration of the mass gathering may be long, as for some festivals or a displaced population in a refugee camp. It may be transitory or recurrent, in association with public transport, repeating sports events, or temple visits.

Mass gatherings are associated with public health challenges, including an increased risk of communicable disease transmission,^2^ although they can also have positive health effects, ranging from feelings of well-being and belongingness that can last for a long time,^3–5^ to beneficial economic effects. The benefits of each mass gathering must therefore be weighed against any increased risk of communicable disease and the availability of effective disease prevention measures.

The risk of communicable disease transmission increases in crowded conditions, where there is close contact with numerous individuals, and where there is a mixing of people from geographical areas with different disease endemicities.^1,2^ During mass gatherings, this risk is exacerbated by shared accommodation, compromised hygiene practices, and other behavioral factors, such as smoking, sharing food, and poor cough etiquette.^1,6^ The appetite for seeking healthcare is often low in this context, leading to continued infection exposure. Once an outbreak is recognized, there are healthcare burdens related not only to patient care but also to the impact on local resources, and potential exposure of the wider society to the pathogen. Additionally, for bacterial pathogens, increased antibiotic prescription and chemoprophylaxis of close contacts raise the risk of the emergence of drug-resistant strains.^7^

The planning process for mass gatherings therefore needs to include a risk assessment and the introduction of measures to prevent and control communicable diseases.^1^ For example, millions of people usually attend the annual Hajj in Mecca, Saudi Arabia. Free healthcare is provided to all attendees and public health teams assess arriving pilgrims, check their immunization status, and administer recommended prophylactic medicines.^8,9^ Different communicable diseases are monitored, including invasive meningococcal disease (IMD) in response to previous IMD outbreaks among pilgrims and close contacts.^10^

IMD is primarily transmitted via direct contact or through the dispersion of respiratory droplets from an infected to a susceptible individual.^11,12^ Incidence estimates vary widely among countries because of diverse standards of IMD surveillance although it is recognized that its incidence is highest among children younger than 1 year and adolescents or young adults.^7^ Carriage prevalence of *Neisseria meningitidis* increases throughout childhood, with estimates suggesting a peak of 24% in late adolescence.^13^ Although a very small proportion of carriers develop IMD, meningococcal carriage represents the first step for disease transmission.^14^ The onset of disease is often rapid, with case fatality rates of 4–20% despite appropriate treatment,^15^ and serious long-term sequelae in up to 20% of survivors.^16,17^ Diagnosis of IMD therefore needs to be accompanied by prompt treatment.^18^ Prevention strategies, most notably vaccination, are extremely effective in controlling meningococcal disease,^19^ while mass chemoprophylaxis can provide temporary protection to recipients during outbreaks.^20^

**Figure 1.**
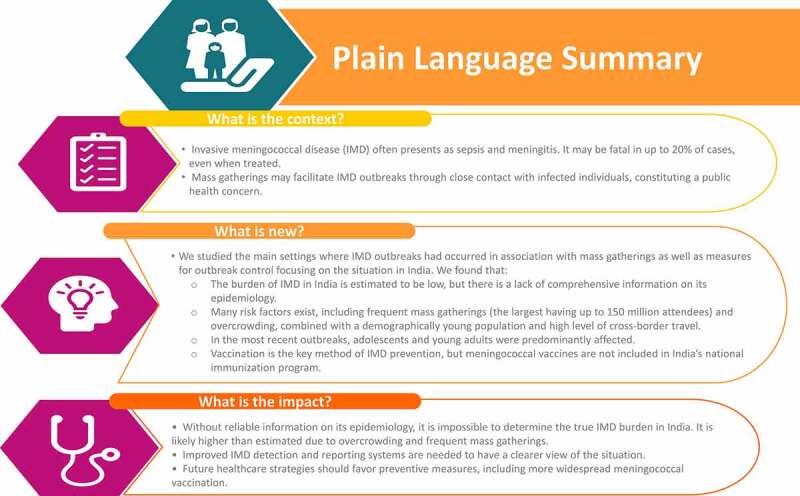
Plain Language Summary

This article provides a narrative review of the main settings where IMD outbreaks have occurred in association with mass gatherings. We then focus on the situation in India, where mass gatherings and overcrowding are common for various socio-demographic reasons. A comprehensive review of the burden of meningococcal disease in this country has been published recently.^21^ We highlight the difficulty in determining the current epidemiology of IMD in India and outline the measures used to contain outbreaks, presenting the case for adjusting the existing public health policy toward this vaccine-preventable disease (Figure 1).

## Meningococcal disease outbreaks related to mass gatherings

Table 1 lists the main settings, with select examples, where IMD outbreaks have occurred in association with crowded conditions. Religious pilgrimages are a well-known example, particularly outbreaks associated with the Hajj and Umrah mass gatherings in Saudi Arabia.^10^ The first reported international meningococcal outbreak following the Hajj occurred in 1987, when a meningococcal serogroup A outbreak spread rapidly among pilgrims, Saudi residents, and was exported internationally via colonized pilgrims returning home and infecting close contacts.^22^ The following year, immunization with the bivalent meningococcal AC polysaccharide vaccine became mandatory for all pilgrims entering Saudi Arabia. In the 2000/2001 season, two major Hajj-related meningococcal serogroup W outbreaks occurred. After the 2000 Hajj, over 400 serogroup W IMD cases were reported in Hajj pilgrims and their close contacts from 16 countries, followed by a smaller outbreak in 2001.^22^ In 2001, chemoprophylaxis was introduced for all local pilgrims before returning to their families and in 2002, immunization with a quadrivalent meningococcal ACWY vaccine became mandatory. Combined with strict disease surveillance, this strategy of meningococcal vaccination has been exemplary in preventing further IMD outbreaks.^10^

**Table 1.** Main settings and selected examples where invasive meningococcal disease outbreaks have occurred in association with a mass gathering or other over-crowded setting

Setting	Example and reference	Country of origin	Year of outbreak
Religious pilgrimage	Hajj and Umrah^22^	Saudi Arabia	1987, 1992, 1997, 2000, 2001
Ceremonial gathering	Funeral^23^	Liberia	2017
Sporting event	International youth football tournament^24^	Belgium	1997
Other gatherings of adolescents/young adults	World Scout Jamboree^25^	Japan	2015
	School bus^26^	Australia	2005
	University campus^27^	USA	2013–2018 (10 outbreaks)
Refugee population	Refugee camp^28^	Italy	2015/2016
Military personnel	Military camp^29^	India	2006

IMD outbreaks have also been reported in association with ceremonial mass gatherings, such as funerals, as reported in Liberia, which is outside of the African meningitis belt (the region in sub-Saharan Africa with a very high incidence of meningococcal disease) and therefore not a country where meningococcal disease was suspected immediately.^23,30^ Across three counties, 31 cases of unexplained illness and death were reported among individuals who attended a two-day funeral in 2017. Subsequent laboratory testing, using confirmatory direct real-time polymerase chain reaction (PCR) assays, detected *N. meningitidis* in 14 patients, with serogroup C identified in 13 patients. Further, *N. meningitidis* was identiﬁed in all 11 fatal cases with specimens available.^30^ This experience illustrated the importance of rapid laboratory confirmation where the etiology of the outbreak was unknown.

The second peak of IMD incidence, after the first in infancy, occurs in adolescents and young adults,^31^ usually when individuals are living in close quarters, such as in conjunction with mass gatherings.^32^ This occurred in association with the World Scout Jamboree held in 2015 in Japan, involving over 33,000 participants from 162 countries.^25^ On their return home, five IMD cases were reported among Scottish and Swedish scouts, as well as an additional secondary case in a parent, all of which were caused by meningococcal serogroup W, which is rarely detected in Japan.^25,33^ This outbreak, therefore, highlighted the risk of IMD outbreaks in mass gatherings held in low-incidence settings. There have also been reports of outbreaks among adolescents and young adults that are likely to have been related to other crowded situations, such as school transport^26,34^ and college residence halls and social venues.^27,35,36^ For example, 10 university-based outbreaks occurred in seven USA states during 2013–2018, involving between two and nine cases in each outbreak, and lasting up to 379 days.^27^

Sporting events present another setting with an increased likelihood of crowding and close proximity among people for prolonged periods. IMD outbreaks have been reported following various sporting events in Europe.^24,37–39^ For example, following an international youth football tournament in Belgium in 1997 with over 1,300 participants,^24^ 11 cases of meningitis or sepsis were reported in four countries (Denmark, the Netherlands, Germany, and Belgium), all caused by meningococcal serogroup C. Eight of the cases were adolescent, and three cases were aged 29–39 years, one of whom died. The response to this outbreak involved international cooperation, although control measures varied according to national guidelines, emphasizing the need for greater consistency. These outbreaks also highlight the need to consider large sporting events as mass gatherings in national IMD prevention guidelines.

Other meningococcal outbreaks related to crowded conditions concern refugee populations, as reported in Italy, Turkey, Uganda, and the Democratic Republic of Congo.^28,40–43^ The military is also at higher risk of IMD because of communal and crowded living quarters combined with deployment or training in regions with different meningococcal serogroup epidemiology.^44^ Various outbreaks have been reported,^29,44,45^ pointing toward the need to offer vaccination to military recruits at commencement of training.^44^

## Mass gatherings in India

**Figure 2.**
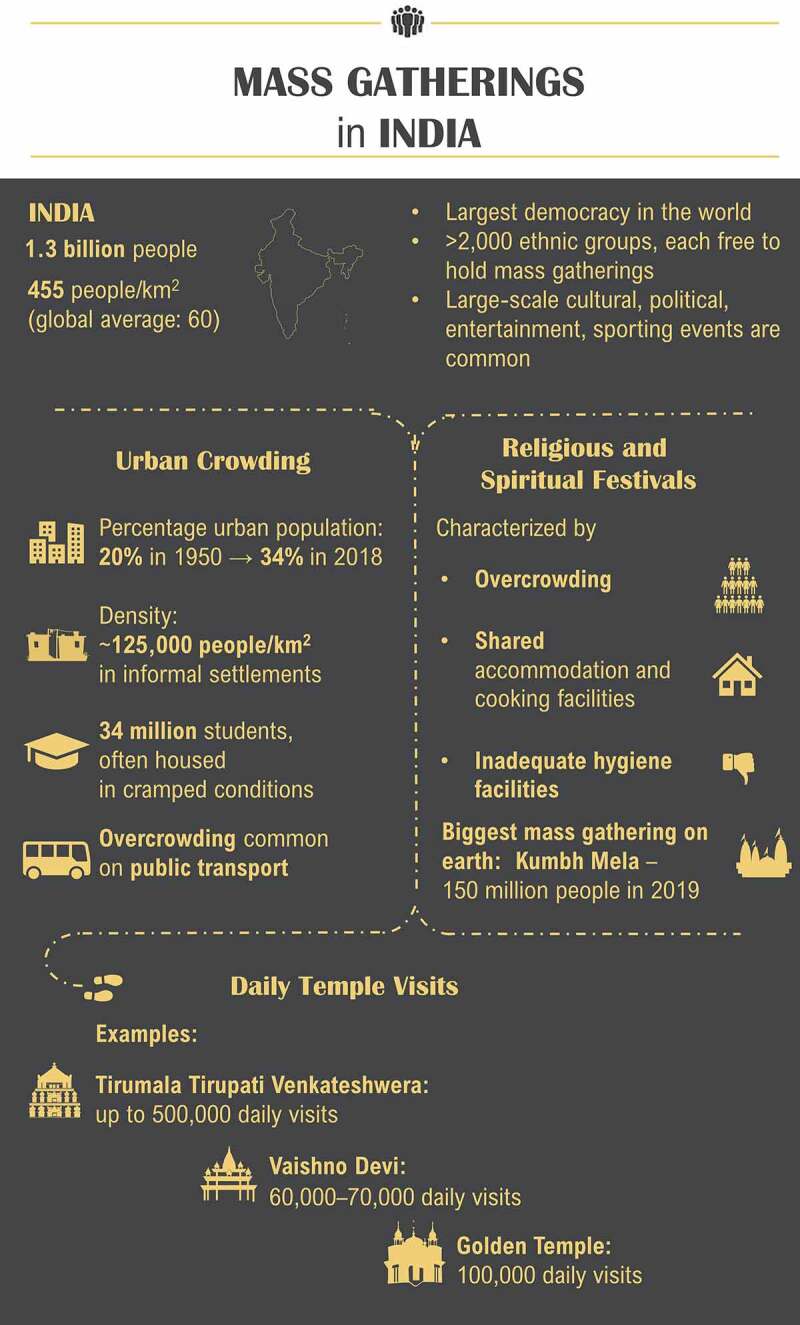
**Mass gathering and over-crowding settings in India**.^46^**^,^**^47^

India is the world’s largest democracy, with more than 2,000 ethnic groups^46^ and an estimated population density in 2018 of 455 people per square kilometer of land^47^ (Figure 2). Because of India’s democratic constitution, ethnic diversity, and dense population, mass gatherings are regular occurrences. These include religious and spiritual ceremonies relevant to each ethnic group, and other cultural, political, entertainment, and sporting events.

The biggest human mass gathering on earth is held in India: the Hindu festival, Kumbh Mela, a three-month-long event held every three years in one of four different cities (Allahabad, Nasik, Haridwar, and Ujjain) in the north of India.^48^ An estimated 150 million people attended the 2019 Kumbh Mela.^49^ Pilgrims travel to the Kumbh Mela by different means (by air, road, rail, and foot) from within India, making it difficult to monitor the health of visiting pilgrims.^48^ The pilgrims generally stay in a make-shift city where there is overcrowding, sharing of accommodation and cooking facilities, and hygienic practices are often compromised. All these factors have implications for public health, increasing the risk of disease transmission.^48,50^

Temple visits in India can involve mass gatherings that occur on a daily basis. For example, the Tirumala Tirupati Venkateshwera temple in south India has daily visitor numbers of around 100,000, increasing to 500,000 during Hindu festivals. Vaishno Devi, a temple situated in a mountain cave in the northern state of Jammu, has approximately 60,000–70,000 visits daily. The Golden Temple in the state of Punjab, the holiest shrine for Sikhs, attracts an estimated 100,000 people per day, which can double during the festival of Gurupurub and Baisakhi. During visits, people sit together to eat, with up to 5,000 people served at each sitting from a ‘mega kitchen’.

As well as mass gatherings, overcrowded situations occur frequently in India, often in relation to living conditions and the movement of people in cities. Urban informal settlements are densely populated residential areas characterized by insufficient access to adequate water and sanitation services and households that frequently edge city drains, railway tracks, and low-lying flood-prone areas. The population density varies but is estimated to exceed 125,000 persons per square kilometer in the largest urban informal settlements in India, which are in Mumbai and Hyderabad.^51^ Moreover, the proportion of residents in urban settlements increased from under 20% in 1950 to 34% in 2018, and projections indicate near doubling of the urban population in India between 2018 and 2050, with an additional 416 million urban dwellers.^52^ This growth is largely attributed to rural-to-urban migration. Since migrants are often younger, on average, than the populations living in areas of origin or destination, migration tends to lower the average age in destination areas.^52^

Another common setting in India of overcrowded living conditions is student accommodation. Globally, India has one of the youngest populations, with approximately 58% aged under 30 years.^53^ More than 34 million students are enrolled in courses at universities across India and most live in small shared spaces in apartments and hostels.^54^ A review of meningococcal carriage in high-risk settings found that university students had the highest rates of carriage (up to 71% but more usually around 10–30% carriage), although most data are from the USA or Europe.^12^ Nevertheless, overcrowded student accommodations combined with social behaviors that encourage close physical contact suggest there is likely to be an elevated risk of meningococcal transmission among students in India. However, there has been only one study of meningococcal carriage among college students in India, which showed nasal carriage of 1.5% among 274 students from a single center in 2014.^55^ Since this study was carried out in one north Indian setting, this finding was not generalizable to the whole country. Additionally, the effect of living in close proximity was not studied as the students were only surveyed at one timepoint; it was planned to re-test them after six weeks but this was not possible as the institutions had to close due to flash flooding.

The rapid growth of India’s urban population has put enormous strains on all public transport systems, with overcrowding common on trains and buses.^56^ At peak times, passengers are packed together, and trains and buses routinely carry up to two times the actual capacity, forcing people to be in close proximity to each other in enclosed spaces. Moreover, the risk of exposure to meningococcal infection that originated outside of India is likely to have risen in recent years with increased international travel. In 2018, there were 10.6 million foreign tourist arrivals in India, an increase of 5.2% on the previous year, and 26.3 million Indian nationals made a cross-border journey, which had an annual growth rate of 9.8%.^57^

## Epidemiology of meningococcal disease in India

IMD is recognized as a notifiable disease in India and a recent literature review concluded there is a significant burden of meningococcal disease-related complications in this country.^21^ The review also found that meningococcal disease is increasingly reported among adolescents and adults, with large outbreaks described in these populations, the most recent of which occurred in Meghalaya^58^ and Tripura.^59^ The Meghalaya outbreak occurred from January 2008 until June 2009 with 110 cases aged up to 18 years and seven deaths from one center as per the study by Hazarika et al.,^58^ whereas the total number of cases in the state were 2100 with 260 deaths. The Tripura outbreak, which occurred from January to August 2009, included 285 suspected and confirmed cases and 62 deaths, and most cases were aged 20–30 years. In both outbreaks, IMD was caused by meningococcal serogroup A.^58,59^ Meningococcal disease in India appears to be caused almost exclusively by serogroup A, although serogroups B, C, W, and Y have also been documented.^21^ Further epidemiological information is also available from the military in India. In 2005–2010, the Indian Armed Forces had an average attack rate of 9 or 10 cases of meningococcal disease per year and the *N. meningitidis* carrier rate among recruits was estimated in the 1990s to be 12%.^60,61^ The largest outbreak occurred in 2006, in which 17 cases were reported among a group of soldiers, with disease caused by meningococcal serogroup A.^44^

The background incidence of IMD in India has been estimated to be low, with *N. meningitidis* responsible for 1.9% of the meningitis cases, although this estimation is based on data gathered from 1950 to 2007.^62^ Moreover, as discussed in detail previously,^21^ there is a lack of comprehensive information on the epidemiology of meningococcal disease, with few data from the last decade. Small outbreaks are likely to be unreported, particularly those in rural areas, and the true magnitude of larger-scale outbreaks is considered to be underestimated.^62,63^ This underestimation occurs because, although IMD is a notifiable disease, disease surveillance is not enforced and reporting is passive only.^21^ The focus is therefore on disease management rather than the collection of surveillance data.^63^ Most of the available epidemiological data were collected during outbreaks^64^ and case reporting and disease monitoring activities were discontinued once the epidemic had resolved.^63^ Moreover, as in other resource-limited countries,^65^ diagnosis has been most commonly via bacterial culture rather than PCR. Culture-conﬁrmed diagnosis of IMD is often hindered by early antibiotic treatment,^66^ which is highly prevalent in India: between 2000 and 2015, antibiotic consumption doubled in India, to 6.5 billion defined daily doses.^67^ Combined with resourcing constraints on government reference laboratories for processing clinical samples, these factors have led to a lack of reliable data on IMD incidence and the prevalence of meningococcal carriage in India.

## Measures to prevent and contain meningococcal disease outbreaks in India

The measures used to contain IMD outbreaks in India are summarized in Table 2.^64^ These are implemented by a rapid response team, consisting of epidemiologists, microbiologists, and medical professionals, which is deployed for immediate action, although more remote areas of the country often face a lag time in the response. If diagnostic facilities are not available locally, as is typical in remote areas, patient samples are sent to the National Center for Disease Control (NCDC) for diagnostic testing.

**Table 2.** Actions recommended to be undertaken immediately after confirmation of a meningococcal disease outbreak in India.^64^ These actions are implemented by a rapid response team, typically composed of an epidemiologist, microbiologist, and medical professionals

Active case surveillance
Early diagnosis and prompt treatment
Chemoprophylaxis of close contacts (household members, HCPs)
Foster disease awareness within the community, including the need to seek medical help and avoid crowded places
Respiratory isolation of patients for 72 hours
Reactive vaccination of close contacts, HCPs, and high-risk groups, or mass vaccination in the affected area, depending on the extent of the outbreak

HCP, healthcare professional.

Chemoprophylaxis of close contacts is recommended to decrease the risk of transmission,^18^ usually with ciprofloxacin or, alternatively, rifampin or ceftriaxone. However, antimicrobial resistance is a major threat to public health, particularly given the high prevalence of antibiotic treatment and misuse in India.^51,67^ Addressing antimicrobial resistance is recognized as one of the top 10 priorities for collaborative work between the Indian Ministry of Health and the WHO.^68^ A national action plan on antimicrobial resistance has been implemented since 2017, including the aim of reducing infection by effective prevention or control measures.

The WHO recommends large-scale meningococcal vaccination programs in countries with high or moderate endemic rates of IMD and in countries with frequent outbreaks.^69^ A growing number of countries have included vaccination against meningococcal disease in their national immunization programs.^21^ In India, routine vaccines are administered via the state-sponsored Expanded Program on Immunization (EPI) and meningococcal vaccination is not included in the EPI list. Consequently, meningococcal vaccination is mainly limited to outbreak control. Since the immunological response to vaccination is not immediately protective,^70^ the effectiveness of this reactive approach is limited if the outbreak is abrupt and short-lasting, or if there is a delay in reporting cases to the NCDC, which may occur in remote parts of the country.^63^

There are however circumstances where meningococcal vaccination is administered outside of outbreak control. The Indian Academy of Pediatrics (IAP) recommends vaccination of children with high-risk conditions, such as terminal complement component deficiencies or functional/anatomic asplenia, and persons with HIV infection.^64^ Beyond this, a quadrivalent ACWY vaccine is given to all travelers to Mecca, in accordance with the Saudi Arabian national policy, within 3 years of travel^22^ and meningococcal vaccination is recommended for those studying abroad and travelers to countries in the African meningitis belt.^64^ In 2012, an immunization program with a quadrivalent polysaccharide vaccine was introduced in India for all military cadets and recruits.^63^ Currently, vaccination of military personnel is recommended but is not mandatory.

The IAP recommends conjugate vaccines rather than polysaccharide-only meningococcal vaccines because polysaccharide-only vaccines are T cell-independent and so are only effective for short-term protection in children older than 2 years and adults.^69,71^ Conjugate vaccines elicit longer-term protection from birth and induce immune memory^72,73^ and may prevent the acquisition of meningococcal carriage.^73^ A polysaccharide bivalent (serogroups AC) vaccine and polysaccharide or conjugate quadrivalent (ACWY) vaccines are licensed against IMD in India. Polysaccharide vaccines are used most widely via the EPI or where vaccination is mandatory, while conjugate vaccines are more commonly used in the private sector. The most recent example of mass vaccination by using the polysaccharide AC meningococcal vaccine was during the Meghalaya and Tripura outbreak in 2008–2009. Any vaccine not included in the EPI schedule is purchased privately^74^ and there is little focus in India on immunization beyond early childhood, with no published national immunization guidelines for adults.^75,76^

## Meningococcal disease prevention and control policies for mass gatherings

The WHO has outlined key considerations for preventing and controlling infections associated with mass gatherings, as summarized in Table 3.^1^ These practical suggestions are based on experience from various events, highlighting the multitude of logistical issues that are relevant for improving the public health response to both planned and unplanned mass gatherings. Briefly, before each event, there needs to be an exchange of information on applied measures and their effects, experience and consultations with previous event organizers, and an understanding of the risk of infection from the disease endemicity both locally and in participating nations. During the event, procedures should be in place for early detection and response to infection, and, post-event, there should be continued awareness of any communicable disease related to the gathering, which may require international cooperation.

**Table 3.** Practical suggestions provided by the World Health Organization to prevent and control infection during mass gatherings.^1^^.^

**Before the event**
Identify infection risk Identify infection prevention and control (IPC) measures already in place Determine the training status of HCPs on IPC measures and address any shortcomings Examine existing legal framework for implementing appropriate IPC measures Perform cost-effectiveness analysis of IPC measures Assess status of public health service and other services involved in IPC International collaboration to assist risk assessment, share information and experience, and ensure rapid communication and alert about potential threats and access to expertise, laboratory tests, etc. Assess the potential for mitigation measures, including pre-event immunization, assessment of health status before entry, setting up sanitation stations, etc. Assess the impact of more restrictive IPC measures on public opinion, political, economic, psychological consequences, and human rights Develop IPC guidelines according to international recommendations and adapt these to the local situation Train staff involved in events in IPC measures, e.g.,, point of entry staff, security, first aid, environmental health staff responsible for hygiene, HCPs (in hospitals, emergency departments) Regularly check supplies of equipment and utilities to be used in IPC (vaccines, antimicrobials, disinfectants, etc.), to ensure there are sufficient, easily accessible quantities
**During the event**
Ideally, provide free access to healthcare to encourage rapid identification and response to potential infection Provide easily accessible written information to all participants in the languages used by most attendees, taking into account health beliefs and practices and the social context. Populations with low health literacy need to be supported and empowered to act on health information, for example, using visual aids Immunize contacts of infected individuals and monitor the health condition of close contacts Transport cases according to established protocol Isolate individuals suspected to be contagious and quarantine those who have been exposed to communicable disease Provide appropriate responses to infection risk, e.g. easy access to hand hygiene measures, wearing of face masks, encouragement of good cough etiquette, social distancing, and contact avoidance Communicate infection risk with general public, using materials developed before the event
**After the event**
Ongoing situational awareness to ensure any information linked to the spread of communicable diseases is reported rapidly and appropriate response is undertaken. This may involve the international community for a large mass gathering International collaboration after the event, including the exchange of knowledge and experience

HCP, healthcare professional.

In relation to India, its current IMD prevention and control policies need to be viewed in the context of various socio-demographic factors that are likely to increase the risk of IMD further, as described earlier. Half of the population is projected to be urban by 2050, up from one-third in 2018,^52^ and this is accompanied by unplanned and crowded settlements, poor sanitation, and a high frequency of travel from other regions and countries. A variety of travel means are used to reach mass gatherings, which makes it difficult to monitor the immunization status and health of attendees, a key part of the WHO recommendations.^1^ Monitoring is easier for mass gatherings where the majority of attendees arrive by air, such as the Hajj.^48^ The relatively young age of the general population in India is another factor to be taken into account, given the peak in IMD incidence in the adolescent/young adult age group.^7,62^

Since IMD is a vaccine-preventable disease,^73^ these risk factors could be addressed with routine meningococcal vaccination but currently however, only vaccination during an outbreak, and vaccination of certain high-risk groups, students studying abroad, and travelers to the Hajj and sub-Saharan Africa, are sponsored by the Indian government.^63,64,71^ The decision to not include meningococcal vaccination in the EPI is based on a perceived low burden of IMD^64^ but, as already discussed, there is a lack of accurate data on its epidemiology. There is therefore a need for better IMD surveillance in India and, as concluded by attendees of the Global Meningococcal Initiative India, this surveillance needs to integrate disease reporting from both the public and private sectors.^63^

## Conclusions

In conclusion, without reliable surveillance data and laboratory confirmation with strain characterization, it is impossible to determine the true IMD burden in India but it is probably safe to presume that it is higher than estimated. IMD risk factors abound in India, with frequent mass gatherings and overcrowding, combined with a demographically young population and high level of travel within India and internationally. Avoidance of mass gatherings and overcrowding is extremely difficult, as is the logistics of monitoring the health of those attending large events. Moreover, with the inclusion in the national immunization program of the *Haemophilus influenzae* B vaccine and pneumococcal conjugate vaccine, the etiology of meningitis in India may shift toward *N. meningitidis*.^77^ Future healthcare strategies are likely to favor strengthening meningococcal disease surveillance and introducing preventive measures that simplify outbreak control and avoid increasing the threat of antimicrobial resistance to public health. Given the abundance of meningococcal disease risk factors and the potential life-threatening nature of IMD, there is a rationale for offering vaccination against meningococcal disease to the wider community in India.

## Abbreviations

EPI, Expanded Program on Immunization; IAP, Indian Academy of Pediatrics; IMD, invasive meningococcal disease; NCDC, National Center for Disease Control; WHO, World Health Organization.
